# Factorial Designs Help to Understand How Psychological Therapy Works

**DOI:** 10.3389/fpsyt.2020.00429

**Published:** 2020-05-14

**Authors:** Edward R. Watkins, Alexandra Newbold

**Affiliations:** College of Life and Environmental Sciences, University of Exeter, Exeter, United Kingdom

**Keywords:** factorial, mechanism, psychotherapy, specific factors, common factors, cognitive behavioral therapy

## Abstract

A large amount of research time and resources are spent trying to develop or improve psychological therapies. However, treatment development is challenging and time-consuming, and the typical research process followed—a series of standard randomized controlled trials—is inefficient and sub-optimal for answering many important clinical research questions. In other areas of health research, recognition of these challenges has led to the development of sophisticated designs tailored to increase research efficiency and answer more targeted research questions about treatment mechanisms or optimal delivery. However, these innovations have largely not permeated into psychological treatment development research. There is a recognition of the need to understand how treatments work and what their active ingredients might be, and a call for the use of innovative trial designs to support such discovery. One approach to unpack the active ingredients and mechanisms of therapy is the factorial design as exemplified in the Multiphase Optimization Strategy (MOST) approach. The MOST design allows identification of the active components of a complex multi-component intervention (such as CBT) using a sophisticated factorial design, allowing the development of more efficient interventions and elucidating their mechanisms of action. The rationale, design, and potential advantages of this approach will be illustrated with reference to the IMPROVE-2 study, which conducts a fractional factorial design to investigate which elements (e.g., thought challenging, activity scheduling, compassion, relaxation, concreteness, functional analysis) within therapist-supported internet-delivered CBT are most effective at reducing symptoms of depression in 767 adults with major depression. By using this innovative approach, we can first begin to work out what components within the overall treatment package are most efficacious on average allowing us to build an overall more streamlined and potent therapy. This approach also has potential to distinguish the role of specific versus non-specific common treatment components within treatment.

## Introduction: The Need to Understand How Psychological Therapies Work

Psychological treatments for mental health disorders have been robustly established as proven and evidence-based interventions through multiple clinical trials and meta-analyses (1–3). Nonetheless, there is a pressing need to further improve psychological interventions: even the best treatments do not work for everyone. Many patients do not have sustained improvement, and treatments need to scaled up to tackle the global burden of mental health (4). For example, psychological treatments for depression only achieve remission rates of 30%–40% and have limited sustained efficacy (at least 50% relapse and recurrence) (1, 5). Further, it is estimated that current treatments, if delivered optimally, would only reduce the burden of depression by one third (6). As such, psychological treatments for depression need to be significantly enhanced.

One pathway to improving the efficacy and effectiveness of therapies is to develop our understanding of how complex psychological interventions work. Despite determining that a number of psychological treatments are effective, for example, cognitive-behavioral therapy (CBT), we still do not know how psychological treatments work. There is little evidence on the precise mechanisms through which psychological treatments work or what are the active ingredients of treatments (7–10), especially for disorders involving general distress such as depression and generalized anxiety disorder. Historically, there has been little progress in specifying the active ingredients of CBT for depression, and as a consequence, there have been no significant gains in the effectiveness of CBT for depression for over 40 years.

Resolving the active mechanisms and active ingredients of psychological interventions has been repeatedly identified as a major priority for research (4, 7, 10, 11). For example, the Institute of Medicine (2015) highlighted the need to identify the key elements of psychosocial interventions that casually drive its effects (11).

To be clear, we distinguish between the active components of therapy, operationalized as the active elements or ingredients within a therapy that produce clinical benefit, which could be therapist-based, client activities, specific techniques, or related to therapy structure and delivery, versus the active mechanisms of the therapy, operationalized as the underlying change processes that causally underpin therapeutic benefit. While active components will necessarily impact on one or more active mechanisms, knowing the most effective components of a therapy is distinct from knowing how this component leads to symptom change [i.e., its underlying mechanism(s)]. For example, in CBT, identifying behavioral activation as an active therapy component does not necessarily confirm that the mechanism-of-action is behavioral as behavioral activation may work through changing cognitions.

Understanding the mechanisms or the active components of psychological treatments are important because either potentially enables the development of more direct, precise, potent, simpler, briefer, and effective treatments. Understanding the active components of a psychological therapy is necessary in order to parse and distil the therapy to focus on what is essential and most engaging to patients.

Psychological treatments are complex interventions, typically made up of multiple elements and components, including the particular content and techniques of the therapy, the interaction between the therapist and patient, the structure of the therapy, and the mode and organization of delivery, each of which potentially acts *via* distinct mechanisms. Therapy is thus a complex multifactorial process. Any or none of these factors could contribute to the efficacy of an intervention, alone or in interaction with the other factors. It is therefore critical to determine the beneficially active, inactive, or inert, and iatrogenic components within an intervention so that the intervention can be honed to become optimally effective, by focusing on the active elements and by removing irrelevant or unhelpful elements (12).

Relatedly, if we know the active mechanisms of an intervention, we may be able to adapt the intervention or develop novel approaches to more directly target this mechanism and, thereby, increase the efficacy of the intervention.

Because of the high prevalence of common mental health problems, there is also a scalability gap because there are not sufficiently available therapists to tackle the global burden of poor mental health (13). It is therefore critical that ways are found to make treatments more efficient, scalable, and easier to train and disseminate. Understanding the underlying components of therapy and being able to remove unnecessary elements may make psychological therapies more effective and more cost-effective by streamlining and simplifying the treatment. For example, the same treatment benefit could be achieved from fewer sessions, enabling a greater volume of patients to be treated for the same volume of therapists. Understanding the critical active components of therapy will also help to adapt treatments for the alternative delivery means that are necessary for increased scalability (for example, to convert for self-help, lay provision, or digital interventions), without losing the core elements needed for efficacy. Understanding how therapy works will also make it easier to effectively train and disseminate therapies, facilitating wider treatment coverage. This understanding may also help to identify moderators of treatment outcome and more effectively personalize therapy to each individual.

## Common Versus Specific Treatment Factors

One key issue with respect to resolving the underlying mechanisms underpinning the efficacy of psychological treatments concerns the question of whether treatment works through specific versus non-specific common factors (8, 14). Specific factors are procedures or techniques arising from the particular therapy approach, such as those typically described in structured treatment manuals, for example, cognitive restructuring in CBT; exposure in CBT for anxiety disorders. Common (or non-specific) factors are those that are hypothesized to be common across all psychological interventions. The most important of these include a positive and genuine relationship between the therapist and patient, engendering positive expectancies and hope in the patient, and a convincing rationale that explains the symptoms experienced and gives credible reasons for the treatment to be helpful (15). There is a long-standing and still unresolved debate between those who propose that psychotherapies mainly work through specific factors versus those who propose that psychotherapies mainly work through common factors.

One argument made in support of common factors is that different specific psychotherapies are generally not found to differ in efficacy, although this does not logically rule out that treatments may work *via* different mechanisms (16). A recent review concludes that there is as yet no conclusive evidence that either common or specific factors can be considered a validated working mechanism for psychotherapy, in other words, the evidence is insufficient to determine the role of either (8).

The relative contribution of common versus specific factors in the efficacy of psychological interventions has important implications for how therapists should be trained, how therapies should be delivered, and for how treatment services should be organized. If the substantive part of the treatment effect is due to common factors, then therapy training should predominantly emphasize therapists learning how to develop a strong therapeutic relationship, develop a rationale etc. In parallel, therapy research should focus on understanding how to strengthen positive common factor effects. However, if specific factors are important then these also need to be emphasized in training and delineated in further research. Furthermore, the increasing importance of specific factors indicates a potentially greater need for discriminating and selecting therapy to match the individual clinical presentation.

## Methodologies to Examine the Mechanisms of Psychotherapy

### Comparative Randomized Controlled Trials

One reason for limited progress in understanding the mechanisms of psychological treatments is the focus on parallel group comparative randomized controlled trials (RCTs). Parallel group RCTs are the gold standard for establishing if an intervention works more than another intervention or against a control and the best means for establishing the relative efficacy of one treatment intervention versus another. However, they are not designed for investigating the specific mechanisms of how interventions work or identifying the active components of therapy. Because comparative RCTs can only compare the overall effects of each intervention package, they are not intended to and unable to provide information about the performance of the individual elements within complex multifactorial interventions. In standard comparative RCTs, all of the multiple treatment components and factors in an intervention package and their hypothetical mechanisms are aggregated and confounded together in the comparison of one treatment versus another. As a consequence, this design is unable to test specific main effects of treatment components nor any possible synergistic or antagonistic interactions between individual treatment components, limiting advances in mechanistic understanding. If an RCT finds one treatment better than another, we do not know which components made a difference; if there is no difference, we do not know whether there are any components that effected an improvement.

This limitation of standard comparative RCTs also applies to their ability to resolve the relative contribution of specific versus common factors. One major issue concerns the difficulty in finding an adequate control arm to compare against a putative active treatment to distinguish the role of specific versus non-specific factors. Some comparative RCTs and meta-analyses have found that one therapy has outperformed another therapy (17, 18), which proponents of specific factors have argued as evidence for specific treatment effects. However, proponents of the common factors model have counter-argued that sometimes the comparison treatments used are not bona fide therapies, defined as viable treatments that are based on psychological principles and delivered by trained therapists, and thus that this is not a fair comparison. When comparisons are made between bona fide therapies, no differences in efficacy are found (19).

Relatedly, other designs have compared an active treatment to a psychotherapy placebo or attentional control on the argument that any differential beneficial effect observed for the active treatment will then be due to specific factors as the effects of the attentional control can only be due to common factors. However, most psychotherapy placebos do not control for all the potential common factors hypothesized in therapy, and thus, any difference found between a placebo and an active treatment could be due to either specific or common factors or some combination thereof (20). For example, it is hard to generate psychotherapy placebos that are exactly matched to active treatments in therapy rationale and credibility, without the placebo itself becoming a bona fide treatment. Similarly, psychotherapy placebos tend to differ from active treatments with respect to the structure of the therapy, for example, the number and duration of sessions, training of therapist, format of therapy, and range of topics covered. A meta-analysis of comparative trials found that there were larger effect sizes found between active treatments and structurally inequivalent placebos than between active treatments and structurally equivalent placebos, for which there were negligible differences (20). These difficulties in finding matched placebo controls or bona fide interventions have limited the conclusions that can be reached about the relative contribution of specific or common factors examined in parallel RCTs.

Attempts have also been made in RCTs to determine mechanisms by examining changes in putative mediators. For example, in trials of CBT, measures of change in negative thinking are examined as a mediator of symptom change. However, these mediational approaches are necessarily limited because they are still indirect and correlational (7). Even if an intervening variable is found to statistically account for the relationship between the treatment and its outcome, this does not provide strong evidence of a mechanism of change, because it does not support a strong causal inference that the mediator influences outcome. In such associations, the mediator may be a proxy to another variable(s) and there may be another unknown or unmeasured variable that is related to both the outcome and the mediator. Ultimately, direct experimental manipulation of the relevant factor is required for strong causal inference, and this is not possible for multiple elements of psychological interventions within a parallel group comparison RCT.

### Component Study Designs

One experimental approach that has been used to examine the specific elements of psychological interventions is the component study (9), in which the full intervention is compared with the intervention with at least one component removed (a dismantling study) or in which a component is added to an existing intervention to test whether it improves outcomes (an additive study) (21). In principle, this approach can enable a strong causal inference that a component has a direct effect on outcome if there is a significant difference in outcomes between the variant of the therapy with a component and the variant without that component.

Nonetheless, there are limitations of component designs. First and critically, the component design does not necessarily test the main effect of a component, that is, the difference between the mean response in the presence of a particular component and the mean response in the absence of the particular component collapsing over the levels of all remaining factors. This can be illustrated with reference to one of the seminal dismantling studies—the dismantling study of CBT for depression by Jacobson and colleagues (22). In this study, patients with depression were randomized to either the full CBT treatment package including behavioral activation, cognitive restructuring to modify negative automatic thoughts, and work on core schema, or to behavioral activation plus cognitive restructuring or to just behavioral activation element alone, with 50 patients in each arm. No significant difference was found between the three versions, leading some observers to suggest that behavioral activation alone is sufficient for the effects of CBT on depression. However, it is important to realize that all versions of the treatment involved behavioral activation: as a consequence, for example, the trial is testing the effect of cognitive restructuring in the context of behavioral activation versus behavioral activation alone. It can only tell us the effect of that component in the context of the other component. Thus, the effects estimated are only the simple effects of each component with the remaining component set to one specific level. For example, for cognitive restructuring, this design only reveals the effect of cognitive restructuring in the presence of behavioral activation. It does not test the main effect of cognitive restructuring, i.e., does the presence of cognitive restructuring have a treatment effect relative to the absence of cognitive restructuring. Similarly, because there is no condition without behavioral activation, it is not possible to estimate the direct main effect of behavioral activation.

Second, the component design assumes that there is no interaction between the components, that is, that the effect of one component is independent of the presence or absence of other components. This may not always be a realistic assumption. For example, it is possible that behavioral activation and cognitive restructuring either complement each other or are antagonistic to each other.

Third, there is a concern that most component studies are not sufficiently powered to detect a difference between two potentially active treatment arms. For example, it has been estimated based on the assumption that a minimally clinically important difference for depression is d=0.24 that a trial would need 274 participants in each condition.

### The Factorial Approach

We propose the use of factorial and fractional factorial designs as an alternative methodological approach to standard comparative RCTs and component designs, which has advantages over both for resolving the active components of psychotherapy. Factorial experiments allow one to explore main effects of factors and interactions among factors (23–27).

Factorial designs systematically experimentally manipulate multiple components or factors of interest. Indeed, factorial designs are commonly used to test the role of different factors simultaneously in experimental psychology. As such, they meet the requirement for delineating active components raised by multiple commentators (8, 10, 14). For example, the Institute of Medicine (2015, p3-10) recently proposed that “determination of which elements are critical depends on testing of the presence or absence of individual elements in rigorous study designs,” which is exactly what a factorial design delivers.

To give a clinical example, if the Jacobson and colleagues dismantling study of CBT for depression was redesigned as a full factorial study, patients would be randomized across three factors [presence or absence of behavioral activation (BA^+^ vs BA^-^); presence or absence of cognitive restructuring (CR^+^ vs CR^-^); presence or absence of work on core schema (CS^+^ vs CS^-^)]. This means that patients would be randomized to be balanced across 8 treatments cells reflecting all of the possible combinations: all three elements (BA^+^: CR^+^:CS^+^); 2 of the 3 elements (BA^+^: CR^+^:CS^-^; BA^+^: CR^-^:CS^+^; BA^-^: CR^+^:CS^+^); 1 of the 3 elements (BA^+^: CR^-^:CS^-^; BA^-^: CR^+^:CS^-^; BA^-^: CR^-^:CS^+^); or none of these elements (BA^-^:CR^-^:CS^-^). This design can test the main effect of each factor as well as their interactions by comparing the mean effects of combined sets of cells against each other. For example, comparing all 4 cells with BA versus all 4 cells without BA tests the main effect of behavioral activation. The difference from the dismantling design is clear because the dismantling design only has 3 of these 8 combinations (BA^+^: CR^-^:CS^-^; BA^+^: CR^+^:CS^-^; BA^+^: CR^+^:CS^+^), which limits it to only testing simple effects.

Factorial designs have been used extensively in engineering to optimize processes. In the last decade, they have been used to good effect in behavioral health, for example, in enhancing interventions for HIV care and prevention (28) and smoking cessation (29, 30). This approach seems well-suited to expanding to the further understanding of psychological treatments and has been recently adopted in several recent trials (31, 32). We believe that factorial designs have advantages for investigating how psychotherapy works that overcome many of the disadvantages noted earlier for comparative RCTs and component trials, as we will outline throughout this paper.

A fractional factorial design is a variation on the factorial design that employs a systematic approach to reduce the number of experimental conditions to allow a more manageable study, at the cost of allowing only main effects and a pre-specified set of interactions to be tested. Fractional factorial designs require the assumption that higher-order interactions are negligible in size, because they are confounded, or aliased, with lower-order effects.

#### The IMPROVE-2 Study as an Example of a Factorial Design

We illustrate the use of a fractional factorial design to identify the active ingredients and mechanisms of an intervention, with respect to a specific example - the IMPROVE-2 study (Implementing Multifactorial Psychotherapy Research in Online Virtual Environments) [see (32) for further detail). The IMPROVE-2 study is a Phase III randomized, single-blind balanced fractional factorial trial based in England and conducted on the internet. Adults with depression (operationalized as Patient Health Questionnaire-9 scores ≥ 10) recruited directly from the internet and from an UK National Health Service Improving Access to Psychological Therapies service were randomized across seven experimental factors, each reflecting the presence versus absence of specific treatment components within internet-delivered CBT, guided by an online therapist (activity scheduling, functional analysis, thought challenging, relaxation, concreteness training, absorption, self-compassion training) using a 32 condition balanced fractional factorial design (2_iv_^7-2^) (see **Table 1**).

**Table 1:** Experimental groups of the IMPROVE-2 fractional factorial design.

Condition	Functional analysis	Concrete training	Compassion	Absorption	Relaxation	Activity scheduling	Thought challenging
1	no	no	no	no	no	yes	yes
2	yes	no	no	no	no	no	no
3	no	no	yes	no	no	no	no
4	yes	no	yes	no	no	yes	yes
5	no	no	no	yes	no	yes	no
6	yes	no	no	yes	no	no	yes
7	no	no	yes	yes	no	no	yes
8	yes	no	yes	yes	no	yes	no
9	no	yes	no	no	no	no	no
10	yes	yes	no	no	no	yes	yes
11	No	yes	yes	no	no	yes	yes
12	yes	yes	yes	no	no	no	no
13	no	yes	no	yes	no	no	yes
14	yes	yes	no	yes	no	yes	no
15	no	yes	yes	yes	no	yes	no
16	yes	yes	yes	yes	no	no	yes
17	no	no	no	no	yes	no	yes
18	yes	no	no	no	yes	yes	no
19	no	no	yes	no	yes	yes	no
20	yes	no	yes	no	yes	no	yes
21	no	no	no	yes	yes	no	no
22	yes	no	no	yes	yes	yes	yes
23	no	no	yes	yes	yes	yes	yes
24	yes	no	yes	yes	yes	no	no
25	no	yes	no	no	yes	yes	no
26	yes	yes	no	no	yes	no	yes
27	no	yes	yes	no	yes	no	yes
28	yes	yes	yes	no	yes	yes	no
29	no	yes	no	yes	yes	yes	yes
30	yes	yes	no	yes	yes	no	no
31	no	yes	yes	yes	yes	no	no
32	yes	yes	yes	yes	yes	yes	yes

Every factor occurs an equal number of times at high and low levels (i.e. balanced) and all factors are orthogonal to each other. Each effect estimate involves all 32 of the conditions in **Table 1**, thereby maintaining the power associated with all participants. This Resolution IV design means that all main effects are aliased with 3-way and higher interactions, and all 2-way interactions are aliased with 2-way and higher interactions, on assumption that non-negligible 3-way interactions are unlikely. In contrast, a standard RCT is aliased for all main effects and interactions of treatment components.

All components involved brief prescribed therapist online support to improve retention and adherence, in which secure online written feedback was provided at the end of each completed module (typically fortnightly), with the option for additional secure messaging between therapist and patient. Therapist feedback highlighted positive steps made, encouraged participants to continue to practice previously introduced components, addressed questions and homework, and pointed out areas to focus on in the next module. Therapists were low-intensity Psychological Wellbeing Practitioners and an experienced clinical psychologist.

The IMPROVE-2 trial used a fractional factorial design to retain the benefits of a factorial design while making the study more logistically manageable and feasible to deliver: this fractional factorial design reduces the total number of conditions from 128 to 32. Each component has two “levels” to be compared in the fractional factorial design: either present or absent, i.e., the respective treatment modules are either provided or not provided in the internet platform. IMPROVE-2 therefore tests the main effects and selected interactions for these 7 components within internet CBT for depression to determine the active ingredients of internet CBT. We first outline the general framework used for this study—the Multiphase Optimization Strategy (MOST)—and then explore the particular benefits and methodological issues of using the factorial design to study psychotherapy.

## The Multiphase Optimization Strategy (MOST)

Within IMPROVE-2, the factorial design is used as one stage within a wider framework for improving interventions—the Multiphase Optimization Strategy (MOST) (33–38) approach. MOST, rooted in engineering, agriculture, and behavioral science, is a principled and comprehensive framework for optimizing and evaluating behavioral interventions (33–38).

MOST consists of three stages: a preparation stage in which the relevant factors and components to be investigated are identified; an optimization stage in which a factorial experiment is used to evaluate the main effects and interactions of each factors; and then an evaluation stage, in which an optimized intervention based on the results of the previous trial is tested in a RCT. MOST has been established to enhance treatments for smoking cessation, with earlier factorial designs identifying active components (29), which were then combined into a novel intervention which outperformed recommended standard care in a RCT (39). MOST is well-validated (29, 30, 34, 40) and recommended within the Medical Research Council Complex Intervention guidelines (41, 42). A key advantage is greater experimental efficiency, with a focus on identifying “active ingredients” versus “inactive” or extraneous components before moving onto large-scale comparative trials, resulting in fewer overall resources required to answer the research questions in the long run than with the traditional approach (43). However, to date, MOST has not been applied to psychological interventions for mental health.

The IMPROVE-2 trial is one of the first attempts to apply the MOST approach to psychological interventions, building on the preparation and optimization phases so far. It incorporates the MOST approach with an internet delivery format for CBT to build in treatment reach, scalability, and increased treatment coverage for the optimized treatment from the start, as the goal is to develop an optimized and scalable evidence-based treatment. Another benefit of using such an internet-delivered therapy is that treatment content can be standardized and fixed, and written therapist responses can be closely demarcated, reducing unwanted “drift” from treatment protocols. This helps prevent potential contamination between different treatment components, which is an important consideration for a factorial design.

### The Preparation Stage in MOST

During the preparation stage, a conceptual model for the intervention is developed, and discrete and distinct intervention components are selected. These components are then pilot tested for acceptability, feasibility, evidence of effectiveness, and ease of implementation, and refined as needed. MOST also involves the identification of the optimization criterion, which is the operational definition of the target change sought that is used to judge the optimal intervention, subject to resource or other constraints. For example, this might be greatest symptom improvement that could be obtained for a particular cost or for a particular duration of treatment.

With respect to the IMPROVE-2 study, a previous feasibility study (IMPROVE-1) established that it was feasible to maintain treatment integrity and fidelity across randomization into multiple treatment conditions and to avoid contamination across treatment conditions. Because the IMPROVE-2 study is focused on determining the ingredients of internet-CBT that are most effective for treating major depression in adults, the operational definition for the optimization criterion was the largest reduction in depressive symptoms, as indexed by using change in scores on the Patient Health Questionnaire-9 score (PHQ-9) (44) as the primary outcome.

### Components Within the Psychological Intervention

A key step within this preparation phase is to identify the components that are to be targeted. When planning a factorial study, the best components to choose are those that are: related to a specific conceptual model; distinct from each other in content, approach or delivery method; have some evidence of efficacy, that can be independently administered, i.e., one component is not dependent on another for delivery; and that are hypothesized to address one or two theoretical mediators. In essence, it is important that components can be distinguished from each other in a meaningful way and that they are conceptually related to different mechanisms.

The elements or components selected can be at different levels of analysis and abstraction. The level selected will depend on the specific question or conceptual model. For example, for CBT, the components chosen could relate to the main hypothesized theoretical mechanisms of change and their associated elements, such as activity monitoring and scheduling and detecting and testing automatic thoughts. Alternatively, the components could relate to lower-level, more discrete elements within the treatment techniques such as the behavioral change techniques outlined in a recent taxonomy (45). These behavioral change techniques include behaviors such as self-monitoring, goal-setting, and feedback, which are common across different CBT components as well as other psychotherapy modalities. Alternatively, the components could relate to process-related aspects of therapy such as whether the intervention is therapist-supported versus unsupported, or structural aspects, such as the frequency of treatment sessions.

IMPROVE-2 illustrates the selection of components to be examined. Consistent with the principles above, the IMPROVE-2 study chose treatment components that were conceptually and operationally distinct from each other, so that each can be evaluated independently. As the first attempt to disentangle the active components within CBT for depression, components were chosen that were clearly distinct and that could be linked to the main theorized mechanisms of action in CBT. These components were operationalized at a relatively high-level (e.g., thought challenging to reflect cognitive theories of change; activity scheduling to reflect behavioral theories of change) rather than in terms of the more localized behavioral change taxonomy because the goal was to determine the core components relating to key theoretical conceptualizations of CBT and to maximize the likelihood of finding a positive effect. If, for example, thought challenging was found to be a strong active ingredient, then further studies could dissect which elements including more specific behavioral change techniques are critical to the effects of thought challenging. Three of the components chosen had been identified as elements for CBT for depression, using a Delphi technique (46): applied relaxation; activity monitoring and scheduling; detecting and reality testing automatic thoughts. A further component—functional analysis—is a mainstay of behavioral approaches to depression including behavioral activation (47). Three components related to recent treatment innovations in CBT derived from experimental research (48, 49), with each hypothesized to specifically target distinct mechanisms arising from different theoretical models: self-compassion, concreteness training, and absorption. The components selected relate to three theoretical accounts of how CBT might work: a behavioral account, a cognitive account, and a self-regulation account.

Three components related to behavioral models of depression and of how CBT works. Depression has been hypothesized to result from a reduction in response-contingent positive reinforcement (50), in which the individual with depression experiences less reward and sense of agency as a consequence of changing circumstances (e.g., loss), poor skills, or avoidance and withdrawal. Within the behavioral conceptualization, *activity scheduling* is hypothesized to increase response-contingent positive reinforcement by increasing frequency of positive reinforcement thorough building up positive activities. This treatment component provides psychoeducation about the negative effects of avoidance, includes questionnaires to help patients identify their own patterns of avoidance, provides guidance on activity scheduling to build up positive activities and reduce avoidance (e.g., breaking plans into smaller steps; specifying when and where to implement activities), and exercises in which participants generate their own activity plans.

In parallel, *functional analysis* seeks to determine the functions and contexts under which desired and unwanted behaviors do and don't occur and, thereby, find ways to systematically increase or reduce these behaviors, by exploring their antecedents, consequences, and variability, and then either alter the environment to remove antecedent stimuli that trigger unwanted behaviors and/or practice incompatible and constructive alternative responses to these antecedents. This approach is based on Behavioral Activation (BA) (51) and rumination-focused CBT (49) approaches to depression. More specifically, functional analysis is proposed to target habitual avoidance and rumination by identifying antecedent cues, controlling exposure to these cues, and practicing alternative responses to them (52).

*Absorption training* is also hypothesized to increase response-contingent positive reinforcement by increasing direct contact with positive reinforcers. Absorption training is focused on teaching an individual to mentally engage and become immersed in what he or she is doing in the present moment to improve direct connection with the experience and enhance contact with positive reinforcers. It is designed to overcome the effects of detachment and rumination which can prevent an individual experiencing the benefits of doing positive activities. When delivered within the internet treatment, patients complete a behavioral experiment using audio-recorded exercises to compare visualizations of memories of being absorbed versus not being absorbed in a task, practice generating a more absorbed mind-set using downloadable audio exercises, and identify absorbing activities.

Two components within the factorial design are based on a cognitive conceptualization of depression, in which the negative thinking characteristic of depression, is hypothesized to play a causal role in the onset and maintenance of depression, and, thereby, reducing negative thinking is hypothesized to be an active mechanism in treating depression (53, 54). Central within CBT for depression is the use of thought challenging or cognitive restructuring to reduce negative thinking (55), and this forms one component in the IMPROVE-2 trial. The internet treatment module that delivers the thought challenging component involves psychoeducation about negative automatic thoughts and cognitive distortions, vignettes of identifying and challenging negative thoughts, and written exercises in which patients practice identifying and then challenging negative thoughts using thought records.

The other cognitive-based component involves concreteness training, based on an intervention found to reduce symptoms of depression in a previous RCT (48) and derived from experimental research indicating the benefits of shifting into a concrete processing style (56, 57). Within the IMPROVE-2 trial, the internet treatment module that delivers this component involves psycho-education about depression, rumination, and overgeneralization, a behavioral experiment using audio-recorded exercises to compare abstract versus concrete processing styles, and downloadable audio exercises to practice thinking about negative events in a concrete way. Unlike thought challenging, concreteness training does not test the accuracy or veridicality of negative thoughts but rather trains patients to focus on the specific and distinctive details, context, sequence (“How did it happen?”), and sensory features of upsetting events to reduce overgeneralization and improve problem-solving. *Concreteness training* is therefore hypothesized to specifically reduce the overgeneralization cognitive bias identified as important in depression (53, 58).

The remaining treatment components are hypothesized to directly improve emotional regulation. *Relaxation* is hypothesized to improve self-regulation by targeting physiological arousal and tension. In IMPROVE-2, a variant of progressive muscle relaxation and breathing exercises was used to reduce physiological arousal and tension in response to warning signs, based on trial evidence that this intervention alone reduces depression (48). The treatment component introduces a rationale for relaxation, provides an online relaxation exercise as a behavioral experiment to test if it reduces tension, and a downloadable relaxation exercise.

*Self-compassion training* is proposed to activate the soothing and safeness emotional system, hypothesized to be downregulated in depression (59). Recent research has highlighted the potential benefit of increasing self-compassion in treatments for depression (49, 60–62), although self-compassion has not yet been directly tested within a full-scale clinical trial for patients with major depression. Within this treatment component, patients read psychoeducation about compassion including useful self-statements to encourage and support oneself, complete a behavioral experiment that compares their own self-talk to how they talk to others, try an audio-recorded exercise visualizing past experiences of self-compassion to activate this mind-set and test its benefits, which is downloadable for further practice, and identify activities they would do more of and activities they would do less of to be kinder to themselves.

### The Optimization Stage of MOST: Factorial Experiments and Their Benefits

The second stage of MOST involves optimization of the intervention, typically through a component selection experiment (sometimes called a component screening experiment), using a factorial or fractional factorial design. This factorial experiment is used to specifically determine the individual effects of each component and any interactions between components. It is important to note that this step could involve multiple experiments and an iterative process of further refining the intervention. For example, if the first component screening experiment observed statistically significant moderators of treatment outcome, such as mode of treatment delivery or location of treatment, a further experiment could be conducted in which the moderators are introduced as factors into the factorial experiment so that they are directly manipulated to enable stronger causal inference about their potential contribution to outcome.

#### Advantages of Factorial Design

There are at least four advantages to the use of a factorial design in resolving how therapy works and what its active mechanisms are.

##### Advantage 1: Directly Testing Individual Components and Their Interactions

The factorial experiment provides direct evidence about the effects and interactions of individual components within a treatment package, which is necessary for methodically enhancing and simplifying complex interventions (41). It can test each individual component and determine its main effect. Critically, it can also determine possible interactions between components, which other experimental designs are unable to do. Thus, a factorial design has distinct advantages when one needs to determine whether the presence of one component enhances or reduces the effect of another. This approach enables us to identify the active components of therapy and to select active and reject inactive/counter-productive components or elements. By comparing the presence versus absence of each component, this factorial design can examine the main effect of each component on the primary outcome, for example, testing whether thought challenging reduces symptoms of depression.

With respect to the IMPROVE-2 study, it is important to note that despite the many trials of CBT for depression, no trials have directly tested the main effect of each of the selected treatment components—for example, does thought challenging have a direct effect on reducing depression relative to no thought challenging? This design therefore provides the first fully-powered test of the main effects of these ingredients of CBT for depression. **Table 1** describes the specific combinations of the two-level intervention factors in the experimental design.

To illustrate how the factorial design works, consider **Table 1**. Main effects and interactions are estimated based on aggregates across experimental conditions. For each main effect, half of the study population are randomized to one level of the factor (e.g., in conditions 9–16, 25–32, presence of concreteness training) and half will be randomized to the other level of the factor (e.g., in conditions 1–8, 17–24, absence of concreteness training). Therefore, the main effect of concreteness training can be determined by comparing the average effect of conditions 9–16, 25–32 versus conditions 1–8, 17–24.

Technically, the IMPROVE-2 study is an internet-delivered component selection experiment with seven experimental factors evaluated, each at two levels ((presence, coded as +1 versus absence, coded as -1 of component, effect coded), using a 32-condition balanced fractional factorial design (2_IV_^7-2^). Effect coding is used because it ensures that main effects and interactions are independent.

A full factorial design of seven factors would have required 2^7^ = 128 conditions, which was deemed to be impractical and too complex to program and administer, and thus a fractional factorial design was chosen. For IMPROVE-2, a 2^7-2^ fractional factorial design was chosen, which reduces the number of experimental conditions by a factor of four, down to 32 conditions. While the full factorial design necessarily includes all possible combinations of all factors, within a fractional factorial design the researcher has to strategically and carefully select a subset of the experimental conditions available.

The first consideration when selecting the subset of the experimental conditions is statistical, with a need to maintain a balanced design in which every factor occurs at an equal number of times at each of the two levels, and in which all factors are orthogonal to each other. This necessarily limits the potential configurations of subsets available. These designs can be mapped out using factorial design tables (63) or statistical packages (e.g., PROC FACTEX in SAS).

The second key consideration is to select the subset of experimental conditions that maximizes the ability to estimate the main effects and interactions that are of highest priority for the research question. Typically, estimating the main effects of the intervention components is a priority. For a fractional factorial design, some of the main effects are going to be confounded (typically referred to as “aliased” within the factorial literature) with higher-order interactions, and thus the subset of experimental conditions needs to be carefully selected so that the main effects are only aliased with higher-order interactions that are judged to be less likely to be significant (e.g., 3-way or 4 way-interactions) or of less theoretical interest.

For IMPROVE-2, the selected design allows the estimation of all main effects and several pre-specified 2-factor interactions among the seven intervention factors; in statistical terminology, it is a Resolution IV design because main effects are only aliased with 3-way and higher interactions. This means that if a potential effect is observed for a particular component, technically the observed effect is due to the sum of the main effect itself and the specific aliased higher-order interactions, i.e., the estimated lower-order effect may include contribution from these higher-order effects. For example, the main effect of concreteness is aliased with the 4-way interaction of functional analysis by compassion by absorption by thought challenging, and the 4-way interaction of functional analysis by compassion by relaxation by activity scheduling and the 5-way interaction of absorption by concreteness by relaxation by thought challenging by activity scheduling. Thus, the actual effect observed is due to the sum of the main effect plus the 4-way and 5-way interactions. If this comparison is significant, the most likely explanation is that the presence of concreteness training produces better treatment outcomes than the absence of concreteness training although we cannot rule out in the fractional design that configurations of 4 and 5 components, albeit unlikely, could contribute to this effect. In interpreting the results, the assumption is that the 3-way and higher interactions are highly likely to be negligible, based on extensive research and principles within factorial experiment research (27, 63). Although in most cases this assumption is reasonable, it may not always apply.

In designing the study, several 2-way interactions were pre-specified as being of particular interest, where it was hypothesized that components might interact with each other, and the design was explicitly chosen so that these 2-way interactions were only aliased with 3-way or 4-way interactions, which we typically expect to be negligible. For example, it was hypothesized that activity scheduling and absorption treatment components may have a positive synergistic effect because the former increases the number of positive activities engaged in, whereas the latter increases the potential absorption and connection with these activities. Similarly, it was hypothesized that thought challenging and self-compassion components may have a positive synergistic effect because thought challenging helps individuals to look logically for evidence against and alternatives to negative self-critical thoughts, while self-compassion encourages a more kindly and tolerant approach to tackle self-criticism.

One choice within the design of the fractional factorial is whether or not it includes the experimental condition in which all intervention components are set to the low level or absent, i.e., a no-treatment control. For the purposes of investigating the active ingredients of therapy, this condition is not necessarily required, since the logic of the factorial experiment is not to compare all the conditions directly with each other, as we would in a comparative RCT, but rather to identify the active components by aggregating mean effects across each factor.

For IMPROVE-2, the fractional factorial design explicitly excluded the condition in which participants receive no treatment components. This has several potential advantages. First, it means that there is not a no-treatment or treatment-as-usual condition, so that the design and trial was suitable for use in a clinical service, where it would not be possible or ethical to randomize patients to not receive any active treatment. Second, because all participants are randomized to active treatment, they are more likely to remain engaged in the trial and to not judge that they are receiving the “inferior option” as can sometimes occur for control conditions.

Within the IMPROVE-2 fractional factorial design, all participants were randomized to receive at least one component of CBT and in the majority of cases 3 or 4 components of CBT. Based on the experience of the IMPROVE-1 feasibility study, in which many patients only completed their first few treatment modules, the IMPROVE-2 counter-balanced the order in which the treatment modules delivering each treatment component were received in the internet platform to ensure that each component was received equally often across all participants as patients progressed through the therapy. In this way, the number and order of treatment components was equivalent between the high (presence) and low levels (absence) of each factor. Of course, this leaves open the question of whether the order of receiving treatment components might be important or not: given the iterative nature of the MOST approach, the effect of sequencing treatment components on efficacy could be a further question for a subsequent component screening experiment.

##### Advantage 2: Manipulation of Hypothesized Mechanisms and Examination of Individual Mediators

The factorial design allows research on the working mechanisms and mediators that allows strong causal inference because each factor associated with a hypothesized specific mechanism is manipulated and the effect of manipulating this factor can be tested directly on secondary measures indexing the putative mediator. The design also enables examination of the mediators of each individual intervention component, because each factor is manipulated independently. For example, this design can test whether the presence of a thought challenging component has a main effect on reducing self-reported negative thinking relative to the absence of thought challenging, and whether this change in thinking mediates change in depression.

To maximize this opportunity to test mediators, the IMPROVE-2 trial required all patients to complete a series of self-report questionnaires at baseline and at each follow-up assessment (at 12 weeks and 6 months post-randomization), as well as after each completed treatment module that index all the putative mediators across all the treatment components. For each treatment component, the putative mediator was related to the primary mechanism which each treatment component is hypothesized to most strongly influence, including rumination (5-item Brooding scale) (64) for the functional analysis component, overgeneralization (adapted Attitudes to Self Scale – Revised) (58) for the concreteness component, self-compassion scale (65) for the self-compassion component, negative thinking (Automatic Thoughts Questionnaire) (66) for the thought challenging component; increased behavioral activity and reduced avoidance (Behavioral Activation for Depression Scale Short-form) for the activity scheduling component (67), and absorption and engagement in positive activities, adapted from measures of “flow” for the absorption component (68). Mediational analyses can then be used to test the hypotheses that each treatment component primarily works through the hypothesized mediator, using the analytical approach outlined by Kraemer et al. (69) and modern causal inference methods. In addition, IMPROVE-2 will investigate potential moderation of the treatment components by site, age, sex, severity of depression, co-morbid illness, and antidepressant use. This design enables us to test whether manipulating a particular component influences the underlying process it is hypothesized to change, and whether that process in fact mediates symptom change. By assessing all putative mediators for all components, we can also test whether components influence other processes, e.g., whether components tackling behavior change cognition or vice versa.

##### Advantage 3: Improved Delineation of Specific Versus Common Treatment Factors

The factorial design provides a stronger test of the relative contribution of specific versus non-specific common treatment factors than existing designs. As noted earlier, the majority of control comparisons are inadequate for disentangling specific from non-specific treatment effects because of the difficulty in creating psychotherapy placebos (attentional controls) that match a bona fide psychotherapy for credibility, rationale, and structure. However, the factorial design overcomes this limitation because for any treatment component (e.g., the relaxation component in IMPROVE-2), the aggregate of the conditions where it is present (i.e., **Table 1**, conditions 17–32) are equivalent for treatment credibility, structure, delivery, rationale, therapist contact, therapist content and techniques and therapist allegiance with the aggregate of the 16 conditions where it is absent (i.e., **Table 1**, conditions 1–16), except for the specific treatment component itself. Moreover, these conditions are also matched in aggregate for all the other six treatment components, since these are balanced in the design. The evaluation of the main effect of relaxation involves the comparison of the average effect for the conditions where relaxation is present versus for the conditions where relaxation is absent. This design therefore provides the strongest control condition available and one that is able to disentangle specific from non-specific common treatment factors. More specifically, this approach is a rigorous test of whether there are specific treatment effects arising from particular treatment components in addition to any non-specific factors common across the treatment components. If there is a significant main effect for any component in IMPROVE-2, then this is strong evidence for a specific treatment effect above and beyond all the non-specific common therapy factors present in CBT. The nature of the non-specific factors tested will depend on the specific components compared in the trial design: because IMPROVE-2 exclusively examines components within internet-CBT, it confounds non-specific factors common across therapies (e.g., therapeutic alliance, rationale) and those specific to internet-CBT and common to all components (e.g., self-monitoring; homework). A different study that took components from different treatment interventions could better delineate non-specific effects common to all therapies. This approach would not rule out some contribution of common factors to treatment outcome, as common factors would be matched across the two levels of the factor, but would be definitive evidence for a specific treatment effect. Conversely, if none of the components were found to have a significant main effect (assuming sufficient power), this would suggest that any treatment benefit was due to common factors.

##### Advantage 4: Factorial Designs Are Efficient and Economical

Factorial designs are efficient and economical compared to alternative designs such as individual experiments and single factor designs because they often require substantially fewer trials and participants to achieve the same statistical power for component effects, producing significant savings in recruitment, time, effort and resources (23, 43).

For example, as an alternative to the factorial design used in IMPROVE-2, a research program could investigate each of the components separately in seven individual experiments or conduct a comparative RCT or a component trial (dismantling or additive design). For IMPROVE-2, it was assumed that the smallest Meaningful Clinical Important Difference (MCID) would be a small effect size (Cohen's d or standardized mean difference=.2) for the main effect of an individual treatment component or interaction between components on pre-to-post change in depression. An alpha of 0.1 was chosen as this is recommended for component selection experiments to decrease the relative risk of Type II to Type I error when selecting treatment components; i.e., to avoid prematurely ruling out potentially active treatment components (23, 36). In order to detect a MCID of d = 0.20 with 80% power at α = 0.10 per treatment, a sample size of N=632 was required (NQuery 7.0). Because participants provide at least five repeated measures on the primary outcome, latent growth curve modeling can be used, which was conservatively estimated to reduce sample size by 30% relative to only using first and last time-point as in an Analysis of Covariance, but then numbers were increased to account for estimated 40% dropout attrition post-treatment, giving a required total sample of N= 736 for the fractional factorial design.

However, the same MCID, power and attrition issues apply for all other trial designs. Thus, each individual experiment would need 736 participants to be adequately powered to examine each component: conducting seven separate experiments to investigate each of the seven components would require N= 5,152, or seven times as many participants as the factorial experiment. A parallel comparative RCT to compare each of the components against each other and against a no-treatment control would have 8 arms and require 368 participants per arm, thus requiring N=2,944, or four times as many participants as the factorial experiment. Similar calculations apply for component experiments – for example a dismantling study that compares a full treatment package (all seven treatment components combined), with incrementally dismantled packages, each with a component removed (i.e., all components minus compassion; all components minus compassion and absorption, etc.) would have 7 arms (assuming there is not a no-treatment control), each requiring 368 participants per arm, requiring N=2,576, or 3.5 times as many participants as the factorial design.

Factorial and fractional factorial designs are efficient and economical because rather than making direct comparisons between experimental conditions as in the other designs, the factorial design compares means based on aggregate combinations of experimental conditions. To illustrate within IMPROVE-2, as indicated in **Table 1**, the estimate of the main effect of concreteness training is based on comparing the aggregate of conditions 9–16, 25–32 where it is present, versus aggregate of conditions 1–8, 17–24 where it is absent; the estimate of the main effect of relaxation is based on comparing sum of conditions 1-16 versus sum of conditions 17–32; the estimate of the main effect of thought challenging is based on comparing sum of conditions 1, 4, 6, 7, 10, 11, 13, 16, 17, 20, 22, 23, 26, 27, 29, 32 versus the sum of conditions 2, 3,5,8, 9, 12, 14, 15, 18, 19, 21, 24, 25, 28, 30, 31, etc. In this way all participants are involved in every effect estimate—it effectively recycles each participant by placing each participant in one of the levels of every factor. As such, the full sample size can be used to determine each of the main effects, making this design efficient for power and sample size.

### The Evaluation Stage of MOST

The third stage in MOST is the evaluation of the optimized intervention. An optimized intervention is systematically built from the results of the factorial experiment by including the most active components with strongest effect sizes relative to the pre-specified optimization criterion, but excluding and eliminating weak inert or antagonistic components. This optimized intervention is tested against the standard evidence-based treatment in a parallel comparative RCT. Thus, to be clear, the MOST approach still retains the parallel comparative RCT as the best method to evaluate one treatment package against another, but adds the factorial design as the most efficient means to investigate the treatment components. In this way, the MOST framework uses rigorous design to identify active elements of a treatment, build a potentially better therapy and then test whether it is an improvement on existing active treatments.

IMPROVE-2 has not yet reached the optimized intervention and evaluation stage. Nonetheless, the logic is clear: based on the results of the IMPROVE-2 factorial experiment, a refined internet CBT treatment package would be produced by retaining those treatment components that had the largest effect sizes for depression, and by removing those components that had minimal or even negative effect sizes. Both the Pareto principle and prior MOST studies suggest that there will be variability in the treatment effect sizes of different components and their interactions, that not all components will be active in the therapeutic benefit of CBT, and indeed, that many will have insignificant effect sizes (30). As such, it should be possible to concentrate the therapy elements to make CBT more potent, and as a minimum more effective.

This process also considers any potential interactions between components. For example, if there was a significant positive two-way interaction between two components, such that adding one component to the another produced larger treatment effects than either on their own, then these factors may be added to the treatment package. In contrast, if there was a significant negative antagonistic interaction between two components, such that together the treatment benefit was less than either on their own, the component with the weakest positive main effect would be probably removed from the treatment package.

If an examination of the estimated effect size of the optimized intervention from the component selection experiment looked favorable, then this optimized intervention would then be tested against an established internet CBT for depression treatment package, to test whether these modifications improved treatment outcome. If the optimized intervention looked unlikely to out-perform existing treatments in the modeling of the treatment estimates, or was found to not be superior in a subsequent comparative RCT, then the MOST logic is that further iterations through the three phases are needed. If this approach indicates that some but not all components within internet CBT for depression have a significant effect size in reducing depression, it will lead to the building of better therapies that focus on the active ingredients and discard inert or iatrogenic elements.

## Potential Limitations

The IMPROVE-2 trial is only one illustration of how the factorial approach could be used to delineate the active components of psychological therapies. As is true for any single study, it has specific limitations. First, it is relatively complex in utilizing seven components. This has the advantage of testing multiple putative active ingredients at once but the risk that with this complex design main treatment effects may be diluted. Adequate testing of treatment components in the factorial design requires each component to be delivered with sufficient difference between the presence and absence of the component to provide a fair test of its main effect. Because the components in IMPROVE-2 each reflect exposure to specific treatment content and techniques, this means that participants need to receive a sufficient dose of the respective content and techniques, that is, complete the relevant modules and practice the relevant behaviors. We sought to achieve this by having each component as a distinct module that is completed over several weeks, and whose content and techniques are then referenced and checked and practised in all subsequent modules and explicitly referred to in the subsequent written feedback from the therapist, to maintain their ongoing use. This meant that the “dose” of treatment elements should be comparable to proven internet CBT treatments and sufficient for testing the main effects.

Nonetheless, there are alternative approaches to tackling this issue. One alternative way to increase treatment dose would be to have a simpler design with fewer treatment components that each run over multiple modules. Another alternative is to test process-focused components such as the degree or nature of therapist support (e.g., support versus no support), or structural components such as the frequency of treatment sessions (e.g., weekly or twice weekly), both of which involving keeping therapy content constant. Such designs straightforwardly deliver a sufficient difference between the presence and absence of the treatment component. Of course, the selection of different components necessarily tests different hypotheses as to the active ingredients of therapy. At this point, it remains an empirical question which of these different components most contributes to treatment outcome. Each approach is equally valid. This is why we strongly advocate for multiple factorial trials to test these different dimensions so that we can systematically enhance therapy.

Related to this limitation, IMPROVE-2 used a fractional factorial design, which raises the potential risk of main effects being confounded with higher-order effects. While this risk is deemed to be very low because 3-way and 4-way interactions are unlikely to be significant, a full factorial design would avoid this assumption. A full factorial would be more suitable for designs utilising fewer components.

A further limitation of the IMPROVE-2 design is that all the components utilize a CBT framework and include generic CBT elements such as self-monitoring, planning, homework and homework review, Socratic review, building new activities, collaboration with the therapist, and a common CBT rationale focusing on thoughts and behavior. As such, if we were to find no main effects for any of the treatment components, we could not determine to what extent any treatment benefit observed was due to non-specific effects common across therapies (such as therapist alliance, remoralization) or due to non-specific effects particular to CBT. Nonetheless, this design still provides a better matched control to investigate specific main effects than prior designs and to test if there any specific main effects. Either pattern of findings (identifying one or more specific main effects of treatment components versus no main effects observed) would still be an advance on our current knowledge and could then be further explored further within the MOST framework.

## Discussion

We have reviewed the importance of better understanding the mechanisms and active ingredients of psychological treatments in order to refine, condense, and strengthen the potency and effectiveness of these treatments. We have shown that standard comparative RCTs and component trials have limitations for determining the specific treatment contributions of individual treatment components within a psychological treatment package and for inferring causality concerning treatment mechanisms. We have shown how factorial and fractional factorial trials can overcome these limitations and have the particular advantages of directly testing individual components and their interactions, of examination of individual mediators and experimental manipulation of hypothesized mechanisms, of being able to distinguish specific factors from common treatment factors, and of being economical and efficient with respect to sample size and resources.

This approach has been illustrated with respect to the IMPROVE-2 trial (32), which will provide the first examination of the underlying active treatment components within internet CBT for depression. Understanding the active components of therapy will enhance our understanding of therapeutic mechanisms and potentially enable the systematic building of more effective interventions. The IMPROVE-2 trial has completed the recruitment, treatment and follow-up stages, with 767 adult patients with depression recruited, and statistical analyses underway. It is anticipated that these analyses will significantly extend our understanding of how CBT works. We believe that this innovative approach may provide a useful means to address recent requests for rigorous study designs to determine which elements within psychological interventions are core active components (4, 7, 10, 11).

## Data Availability Statement

The original contributions presented in the study are included in the article/supplementary material, further inquiries can be directed to the corresponding author.

## Ethics Statement

The study protocol for IMPROVE-2 was reviewed and approved by the South West National Research Ethics Committee, NHS National Research Ethics Committee SW Frenchay (reference number, 14/SW/1091, 30/4/2015). The trial sponsor is the University of Exeter, contact person Gail Seymour, Research Manager.

## Author Contributions

EW and AN both designed, prepared, and delivered the IMPROVE-2 study. EW prepared the first draft of the manuscript, AN commented on the draft, and both EW and AN finalized the manuscript.

## Funding

Funding for the IMPROVE-2 study was provided by grants from the Cornwall NHS Partnership Foundation Trust and South West Peninsula Academic Health Research Network. Funding sponsors did not participate in the study design; collection, management, analysis, and interpretation of data; or writing of the report. They did not participate in the decision to submit the report for publication, nor had ultimate authority over any of these activities.

## Conflict of Interest

The authors declare that the research was conducted in the absence of any commercial or financial relationships that could be construed as a potential conflict of interest.
